# Process Analytical Approach towards Quality Controlled Process Automation for the Downstream of Protein Mixtures by Inline Concentration Measurements Based on Ultraviolet/Visible Light (UV/VIS) Spectral Analysis

**DOI:** 10.3390/antib6040024

**Published:** 2017-12-12

**Authors:** Steffen Zobel-Roos, Mourad Mouellef, Christian Siemers, Jochen Strube

**Affiliations:** 1 Institute for Separation and Process Technology, Clausthal University of Technology, Leibnizstraße 15, 38678 Clausthal-Zellerfeld, Germany; zobel-roos@itv.tu-clausthal.de (S.Z.-R.); mourad.mouellef@tu-clausthal.de (M.M.); 2 Institute for Process Control, Clausthal University of Technology, Arnold-Sommerfeld-Straße 1, 38678 Clausthal-Zellerfeld, Germany; Christian.siemers@tu-clausthal.de

**Keywords:** inline concentration measurements, UV/VIS spectral analysis, PAT for monoclonal antibodies, live pooling, peak deconvolution

## Abstract

Downstream of pharmaceutical proteins, such as monoclonal antibodies, is mainly done by chromatography, where concentration determination of coeluting components presents a major problem. Inline concentration measurements (ICM) by Ultraviolet/Visible light (UV/VIS)-spectral data analysis provide a label-free and noninvasive approach to significantly speed up the analysis and process time. Here, two different approaches are presented. For a test mixture of three proteins, a fast and easily calibrated method based on the non-negative least-squares algorithm is shown, which reduces the calibration effort compared to a partial least-squares approach. The accuracy of ICM for analytical separations of three proteins on an ion exchange column is over 99%, compared to less than 85% for classical peak area evaluation. The power of the partial least squares algorithm (PLS) is shown by measuring the concentrations of Immunoglobulin G (IgG) monomer and dimer under a worst-case scenario of completely overlapping peaks. Here, the faster SIMPLS algorithm is used in comparison to the nonlinear iterative partial least squares (NIPALS) algorithm. Both approaches provide concentrations as well as purities in real-time, enabling live-pooling decisions based on product quality. This is one important step towards advanced process automation of chromatographic processes. Analysis time is less than 100 ms and only one program is used for all the necessary communications and calculations.

## 1. Introduction

Inline concentration measurements (ICM) of individual components in a mixture are critical for almost every unit operation in the field of chemical and biotechnological processes. Although most processes are designed to match specific concentration and purity criteria, the actual values still have to be monitored inline or offline [1].

This becomes even more important for batch operations, like chromatography, where concentration and purity vary over time. For such processes, the inline measurement of concentrations and purities become a game winning objective, but often also a major challenge. 

In the field of analytical chromatography, concentration or purity quantifications are usually based on peak areas. Therefore, great effort is put into the chromatographic separation to achieve a baseline separation, if possible. Nevertheless, this is not always achieved. Very closely related components are often especially difficult to separate. The deconvolution of overlapping peaks allows for better results as well as shorter and less expensive separations. 

For preparative chromatography, complete baseline separation is not desirable; on the contrary, it conflicts with process efficiency and economy. The separation process is optimized for a maximum resin utilization and productivity. The product fractionation is often controlled via timed cut points, where the exact moment for the fraction start and end points are derived from earlier experiments and are therefore not directly related to the current chromatographic run. In this case, critical product attributes like the concentration and purity have to be measured offline after the run. Anomalies in elution behavior often result in shifts of the chromatogram leading to large variations from the expected design chromatogram. Thus, the time-based cut points do not match the purity criteria and might lead to a batch failure or require reprocessing. This problem becomes even worse for continuous chromatography processes. Start- and end-pooling criteria based on real-time, online detection of volume and Ultraviolet/Visible light (UV/VIS) absorbance have proven to be very successful in delivering consistent yield and purity. Online concentration and purity identification as presented here will help to shift the process design from timed to data-based fractionation or column switching and therefore give a greater certainty to match purity and yield targets, thus reducing the risk of batch failure. Also, the safety margins for data based fractionation can be smaller, leading to higher yield and productivity.

Furthermore, the knowledge of concentrations of all components online throughout the chromatographic run will help during process design. At the moment, the chromatographic run has to be fractionated into a lot of small volumes and analyzed offline to identify the actual concentration profiles. This is cost intensive as well as time consuming and, in addition, carries a significant risk of product degeneration of sensitive proteins because of long analysis times. The same problem and solution respectively apply for model parameter determination and model validation for a simulation-based design process [2,3].

Hence, a lot of research has been undertaken to achieve a deconvolution of chromatographic peaks. In general, the approaches can be split into inline and offline methods.

For offline or at-line peak deconvolution in particular two different approaches can be found. A classical at-line method is the division of the complete run into small fractions. These fractions are then investigated by a suitable analytical method afflicted with the method specific failure [4,5,6,7,8]. Although analysis time might be rather short, online sampling always results in a rather large response time. Especially the use of gas or liquid chromatographic analysis often has to deal with overlapping peaks itself due to selectivity variances [9]. 

Another offline or delayed online approach is a full mathematical analysis of the chromatogram. This analysis assumes that every peak will follow a mathematical function, e.g., Gaussian or modified Gaussian, and estimates the function parameters. This assumes a Gaussian or modified Gaussian shape might be approximately right for analytical chromatography, but is rather uncommon for preparative separations. The identification of peaks occurs by first, second or higher order derivatives of the chromatographic signal [10,11,12]. 

To identify a component and determine the parameters for its Gaussian function, the peak must be processed to a certain point. In most cases, at least the first inflection point has to be reached. More often, the peak maximum and second inflection point have to be detected already. Hence, this peak deconvolution method cannot be performed in real-time.

For preparative separations, an improvement can be achieved by the use of process modelling. Having a valid process model implemented during process design, the real behavior and elution of the components can be monitored [13,14,15]. 

Spectroscopy is among the most common methods of detection. Hence, major progress was achieved in terms of a multicomponent analysis using ultraviolet (UV), visible (VIS) or infrared spectral data. Mid-infrared spectroscopy (MIR) was used for host-cell protein quantification [16], UV/VIS spectroscopy for determining the protein and nucleic acid content of viruses [17] as well as for a variety of proteins in a multicomponent mixture [18,19,20].

According to the Beer–Lambert law, the UV-extinction E_λ_ of a component at a given wavelength λ is the product of the concentration *c*, the path length d and a component specific coefficient called extinction coefficient *ε*_λ_ as shown in Equation (1) [21,22]:

(1) $$lg\left(\frac{I_{0,\lambda }}{I_{t,\lambda }}\right)=E_{\lambda ,i}=\varepsilon_{\lambda ,i}\cdot c_{i}\cdot d.$$

Technically, this law applies to highly diluted mixtures only. Nevertheless, the deviations are often negligible.

The UV/VIS extinction over the wavelengths, viz. the UV/VIS spectrum, is unique for almost every component. Thus, the sum spectrum of a mixture can be disassembled into the single component spectra. It can be found that the absorbance of a mixture of *n* components sums up from the single component extinctions according to Equation (2) [23,24]:

(2) $$E_{\lambda }=\sum_{i=1}^{n}\varepsilon_{\lambda ,i}\cdot c_{i}\cdot d.$$

A diode array-based UV/VIS measurement provides as many extinction values as there are diodes in the detector, often 256 or 1024. Each diode represents one specific wavelength sector. Hence, Equation (2) can be formulated for each diode leading to a large set of linear expressions. With prior knowledge of the extinction coefficients of each component *ε*_*λ*,*i*_, this set of linear equations can be solved for the concentrations $c_{i}$. This can be done with several mathematical methods like the least-squares or non-negative least-squares (NNLS) [25,26] algorithm. In theory, the experimental effort to measure the extinction coefficients *ε*_*λ*,*i*_, is low. For each single component one injection with known concentration is sufficient. There is no need to calibrate a large set of mixtures with different compositions and concentrations. However, it might be necessary to measure the extinction coefficients *ε*_*λ*,*i*_ for different concentrations of the single components. It can be shown that for higher concentrations, peaks appear in the UV/VIS spectrum that are not visible at lower concentrations. This is due to detector sensitivity only. 

The first example in this paper shows the application of the NNLS-algorithm by measuring the concentrations of three proteins from an analytical ion exchange chromatography.

Another approach for UV/VIS-diode array detector (DAD) based concentration measurements was introduced by Brestrich et al. [18,27,28]. Partial least-squares regression is used to create a statistical model. The PLS regression compresses a set of even highly collinear predictor data X into a set of latent variables T. With these orthogonal latent variables observations can be fitted to depended variables Y. In this case, X is the UV/VIS spectra and Y represent the concentrations. For a better understanding of PLS, see [29,30,31]. 

In both the work of Brestrich et al. [27] and this work, the SIMPLS-algorithm [30] is used. This does not solve the set of linear equations spanned by Equation (2), but creates a statistic model. This model has to be trained by a set of experiments. Contrary to the non-negative least-squares approach, it is not sufficient to only calibrate for single components. The experimental design has to account for different compositions and different concentrations of each component present in the mixture that should be analyzed later. Thus, the amount of experiments increases dramatically with the number of components. Again, the detector sensitivity has a major impact, as does the detector type, age and utilization status. 

The second example in this paper shows the application of the SIMPLS-algorithm for inline and real-time monomer and dimer concentration measurements of monoclonal antibody IgG.

Although the used algorithm is the same, this work differs from other work by a simpler setup. Instead of different programs for each task, only one self-written program is used to do the data acquisition, all calculations, data storage and communication with the programmable logic controller (PLC). Therefore, there is no software bottleneck allowing for very fast measurements. The PLC controls the pumps and valves of a continuous chromatography prototype that was not used in this work. This work is rather a major milestone to achieving a fully automated and self-optimizing system for the prototype, enabling life pooling, purity-based column switching and advanced quality control.

## 2. Materials and Methods

### 2.1. Model Proteins, Buffers and Columns

All experiments with proteins were carried out in 20 mM NaPi buffer at pH 6.0. For ion exchange chromatographic separations, this buffer was used as equilibration buffer A. For elution buffer B, 1 M NaCl was added. For analytical size exclusion chromatography (SEC), a buffer containing 100 mM sodium sulfate and 100 mM NaPi was used at pH 6.6. All salts were obtained from Merck KGaA, Darmstadt, Germany.

For the three component mixture experiments Chymotrypsinogen A, Lysozyme (AppliChem GmbH, Darmstadt, Germany) and Cytochrome C (Merck KGaA, Darmstadt, Germany) were used.

IgG was obtained from our own cell culture with an industrial Chinese Hamster Ovary (CHO) cell line and purified by protein A chromatography prior to the experiments.

Ion exchange separations were performed on prepacked strong cation exchange columns with Fractogel^®^ EMD SO_3_^−^ (M) (5–50, 1 mL, Atoll GmbH, Weingarten, Germany).

Analytical size exclusion columns Yarra^®^ SEC-3000 (3 μm, 300 × 4.6 mm) were obtained from Phenomenex^®^ Inc., Torrance, CA, USA.

Protein A chromatography was performed with PA ID Poros^®^ Protein A Sensor Cartridges (Applied Biosystems, Waltham, MA, USA).

### 2.2. Devices and Instruments

The experimental setup consisted of a standard VWR-Hitachi LaChrom Elite^®^ HPLC system with a quaternary gradient pump L-2130, Autosampler L-2200 and diode array detector L-2455 (VWR International, Radnor, PA, USA). The later was not used for the ICM but for comparative measurements. For the ICM measurements, a Smartline DAD 2600 with 10 mm, 10 µL flow cell from Knauer Wissenschaftliche Geräte GmbH, Berlin, Germany was used. Experimental validations were based on SEC analysis after online fractionation with a Foxy Jr.^®^ from Teledyn Isco, Lincoln, NE, USA.

### 2.3. Inline Concentration Measurements

The inline concentration measurements are based on UV/VIS spectra measured with a diode array detector (Smartline DAD 2600 from Knauer Wissenschaftliche Geräte GmbH, Berlin, Germany) with 256 diodes and a wavelength range of 190 to 510 nm. The selectable bandwidth is 4 to 25 nm. The highest sampling rate is 10 Hz, which is 100 ms [32]. Data collection and analyses were performed by a conventional Windows desktop computer, which uses a standard EIA RS-232 serial port for the communication with the detector. Since the communication between different applications and programs tends to be a major bottleneck, a self-written program was used. An object-oriented, concurrent and class-based programming language [33] was used (Java). Java runs on its own Java Virtual Machine (JVM), which is available for a variety of platforms and computer architectures. Hence, programming in Java follows the “write once, run anywhere” idea. This makes a program written in Java easily applicable on different machines [33]. The ICM application handles the communication between the DAD and the computer, processes the data, displays the results in different charts and stores the data in “comma separated values” (*.csv) files. The user can choose from several algorithms to implement. Amongst those are the least squares, non-negative least squares [25,26] and SIMPLS (a partial least squares variant) [30], the simplex and powell algorithm and some more. It should be noted that these are not only different algorithm, but different approaches. The non-negative least squares algorithm is used to solve the equation system described earlier (Equation (2)). However, PLS provides a statistical model that is related to the spectral data. Which algorithm should be used depends on the complexity of the given sample. As a starting point, the single-component spectra of each component should be compared. In this work, an application for the non-negative least squares and one for the SIMPLS is shown. 

### 2.4. ICM Examples

#### 2.4.1. Protein Test Mixture on Ion Exchange Column under Analytical Conditions with NNLS

To show the power of ICM for analytical separations, a separation of three proteins on an ion exchange column was performed. Fractogel^®^ EMD SO_3_^−^ (M) was used in prepacked 1-mL columns (5–50, Atoll GmbH, Weingarten, Germany). 

The binding buffer A was 20 mM NaPi buffer with a pH of 6.0. For elution, buffer B 1 M NaCl was added. The chromatographic run started with 100% buffer A and proceeded with a 10 CV gradient to 100% buffer B. A flow rate of 1 mL/min was used. This method is meant not to achieve baseline separation, but produce widely overlapping peaks. 

The test mixture contained Chymotrypsinogen A, Cytochrome C and Lysozyme. 10 mg of each protein were dissolved separately in 1-mL binding buffer, leading to three samples with a concentration of 10 g/L each. To identify the differences in UV/VIS spectra, 20 µL of each sample was injected onto the column and measured with the DAD detector at a certain peak height corresponding to 0.01 g/L. 

**Figure 1:**
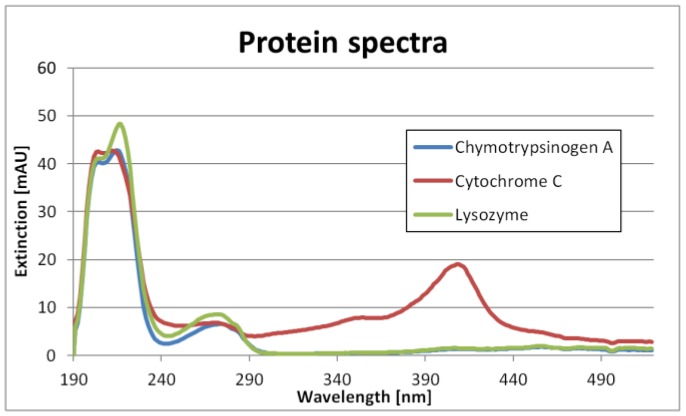
Single-component spectra of Chymotrypsinogene A, Cytochrome C and Lysozyme.

The single component spectra are shown in Figure 1. It can be seen that Chymotrypsinogene A and Lysozyme have similar spectra. Cytochrome C, however, is relatively unique. Nevertheless, the differences are big enough not to need PLS. 

The inline concentration measurements were done by solving the set of linear equations (Equation (2)) for the concentrations with the non-negative least-squares algorithm. Hence, the extinction coefficients *ε*_*λ*,*i*_ for the three proteins were needed.

The extinction coefficients *ε*_*λ*,*i*_ were measured separately for each component. The proteins were dissolved in buffer A separately and injected onto the ion exchange column as described previously. The resulting chromatogram at 280 nm wavelength was converted from extinction to concentration course according to Equation (3):

(3) $$c_{i}\left(t\right)=F\cdot E_{\lambda =280}\left(t\right).$$

The conversion factor *F* was obtained with Equation (4):

(4) $$F=\frac{m_{i,inj}}{\dot{V}\cdot \int_{0}^{t}E_{\lambda }\left(t\right)dt}.$$

Now, the concentration is known for every time point of the chromatogram. Thus, the extinction coefficients *ε*_*λ*,*i*_, are the only unknown variable left in Equation (2). In this work, Equation (2) was solved for the coefficients at a time point corresponding to 0.1 g/L protein concentration. This calibration has to be done for each protein.

One problem of this approach is the superposition of the protein spectrum with any other spectrum of co eluting components. This might result from UV/VIS active compounds in the chromatographic buffer or from leachables/extractables of the column itself. To overcome this problem, a blank run can be done before the actual measurements to perform a baseline correction.

For some components, one might find that different concentrations lead to different spectra. Physically speaking, this is not possible. However, for rising concentrations, peaks in the spectrum might appear that were not present at lower concentrations. These peaks were simply not visible at lower concentrations due to low detector sensitivity. In this case, it is best to analyze the peak at different concentrations, hence obtaining extinction coefficient values at different concentrations. This should be equal except for these cases where detector sensitivity comes into account. For those coefficients, a spline interpolation over the concentration is done.

The actual separation for testing the peak deconvolution was performed with the same column under the same conditions. The test mixture contained all three proteins. 

Quantitative analysis is usually performed based on peak areas. The peak area is a function of the amount of protein injected. To produce the exact same peak areas in the three-component run compared to the single component runs, the same amount of each protein must be injected. Thus, the sample was produced by mixing 0.5 mL of each of the protein solutions used previously. Therefore, this sample has exactly one third of the concentration of each protein compared to the single component samples. Hence, 60 µL were injected to the column.

The peak areas of the single component runs are easily calculated. The corresponding areas from the mixture experiment were obtained by three different methods. First by integrating the concentration curve obtained with the inline concentration measurements. Second, by the perpendicular method. As a typical chromatographic approach, the extinction curve is integrated. The areas between two peaks were divided by a vertical line at the local minima. These areas have to be compared to a calibration curve. The third method is a typical offline peak deconvolution method. The real peak shape is approximated by assuming Gaussian or modified Gaussian behavior. These peaks can then again be integrated and compared to a calibration curve. 

#### 2.4.2. Immunoglobulin G (IgG) Monomer and Dime with SIMPLS

A mixture of IgG monomer and dimer was used to show the potential for pharmaceutical production. Both molecules have almost identical UV/VIS spectra. To distinguish between both is therefore relatively hard. Nevertheless, from a product quality point of view, it is very important to know the concentrations of IgG oligomers.

Since the differences in UV/VIS spectra are low, the SIMPLS algorithm was used. This must be calibrated with a training set of data containing different IgG monomer and dimer concentrations.

The monoclonal antibody IgG came from our own fed batch fermentation of an industrial cell culture line. The cell culture was clarified with centrifugation (3000 g) and filtration (0.2 μm syringe filter, VWR International, Radnor, PA, USA). Afterwards, purification was done with protein A chromatography (PA ID Sensor Cartridges, Applied Biosystems, Waltham, MA, USA). The protein A product peak was then loaded to a size exclusion column (Yarra^®^ SEC-3000, 3 μm, 300 × 4.6 mm, Phenomenex Inc., Torrance, CA, USA). Here, only the upper 50% of the dimer and the monomer peak were fractionated. Hence, the overlapping region of monomer and dimer was not used. After protein A and size exclusion chromatography, no other protein besides IgG and no other contaminates are present. We did not find a fixed equilibrium between the monomer and dimer concentration. However, conversions from one form into the other occur, so that there was no pure dimer or monomer. The concentration in the SEC monomer fraction after some conversion time (overnight) was roughly 0.5 mg/mL monomer and 0.02 mg/mL dimer. The dimer fraction contained 0.2 mg/mL monomer and 0.1 mg/mL dimer.

Out of these two fractions, 11 mixtures were created to calibrate the statistic model. Therefore, different volumetric mix ratios were prepared, starting with 100 vol.% monomer and 0 vol.% dimer. The ratios decreased respectively increased with a 10 vol.% step size to 0 vol.% monomer and 100 vol.% dimer. From eleven mixtures, nine were used as a training set and the other three as validation sets, namely 80 vol.% monomer, 50 vol.% monomer and 20 vol.% monomer.

For each sample, analytical size exclusion chromatography was performed again to get the actual monomer and dimer concentrations.

The experiments itself were performed by injecting 0.1 mL into a small stirred tank of 2 mL total volume. The inlet stream consisted of chromatographic buffer A with a volumetric flow of 1 mL/min. The outlet stream, also 1 mL/min, was directly connected to the Smartline DAD 2600. After the DAD, the stream was fractionated with a Foxy Jr.^®^ (Teledyn Isco, Lincoln, NE, USA) fraction collection module. 0.5 min fractions were taken and this 0.5 mL fractions were analyzed offline with the size exclusion chromatography already mentioned. 

This setup represents a worst-case scenario, since no column or separation takes place. With moderately overlapping peaks, one might enhance your result by applying some mathematical assumptions; this cannot be done here. Furthermore, this experiment simulates conditions one would find in several other, non-chromatographic unit operations within the downstream of monoclonal antibodies, like filtration. This shows the applicability for inline quality control for example in the last filtration step.

## 3. Results and Discussion

The presented inline concentration measurement method shows wide, almost general applicability whenever UV/VIS active component mixtures are involved. In this work, the ability to enhance the accuracy of analytical measurements, and to make real time pooling decisions based on real time data could be shown. For the latter case, the calculation time is of major interest. Since only one program is used for data collection and processing, no time delay could be found throughout all experiments. The UV/VIS diode array detector was run with a sampling rate of 100 ms. The data transfer and all calculations were finished prior to the measurement of the next data point. Thus, one ICM concentration measurement lasts less than 100 ms. 

It should be noted that every method or calibration comes with a systematic error itself. In this case, impurities present in the assumed to be pure calibration mixture could not be detected later on. This applies for example for low level impurities in the protein standard or in the IgG fractions after size exclusion chromatography.

### 3.1. Protein Test Mixture on Ion Exchange Column under Analytical Conditions with NNLS

**Figure 2:**
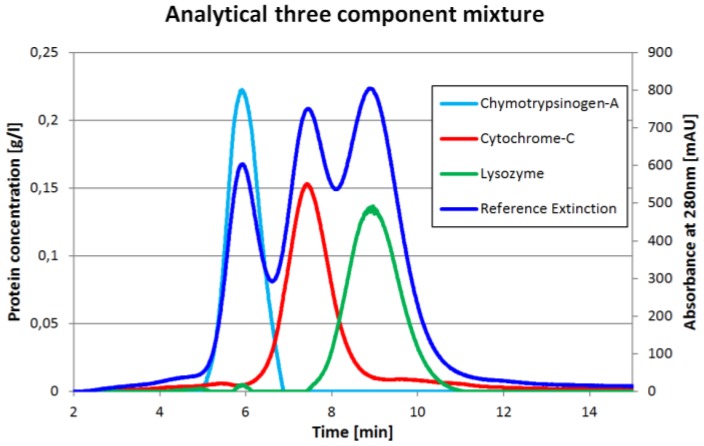
Inline concentration measurement of a three component protein mixture.

One chromatogram and the corresponding inline concentration measurements are displayed in Figure 2. The reference extinction is 280 nm. It is important to understand that the protein concentrations (light blue, red, green) do not have to match the reference extinction plot (dark blue). The proportions would match only if all three proteins had the same extinction coefficient at 280 nm which is obviously not the case. 

**Figure 3:**
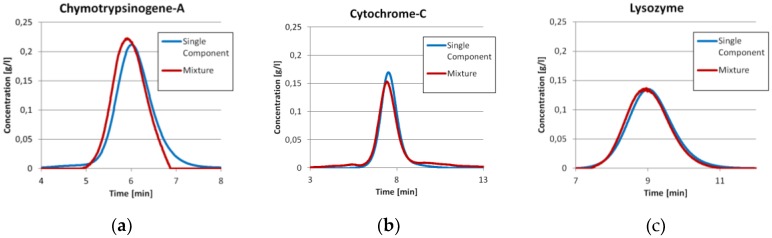
Comparison between the single component injection (blue line) and the deconvoluted peak (red line) of the same component in the mixture for the three proteins Chymotrypsinogene-A (**a**); Cytochrome-C (**b**) and Lysozyme (**c**).

Prior to the injection of the three component mixture, the exact same amount of each component was injected separately as described earlier. The comparisons between the single component injections with the ICM peak of the same component in the three component run are shown in Figure 3. It can be seen that the Chymotrypsinogene-A peak in the mixture is pushed a little bit to an earlier elution. The mean residence time shifts from 6.07 min to 5.96 min. Although, the dilution front of this peak is sharper. This is in good agreement with the displacement theory of chromatography. The Cytochrome-C peak has mainly the same shape in both experiments, but tends to co-elute with both neighboring components slightly. The Lysozyme peak is in both cases more or less the same. For both later proteins, there are only minor changes in the residence time. All values are displayed in Table 1.

The chromatograms in Figure 3 allow for an easy comparison of the total amount of protein in both experiments. Since the ICM method measures concentrations not extinction, the deviations for this method are calculated comparing the total protein amount measured for single component injections to the three component system. This is calculated from feed concentration and injection volume. The mean deviation for the ICM method used for the chromatogram in Figure 2 is 0.02% with a standard deviation of 0.58% compared to −16.99% ± 7.01% for the perpendicular, 17.58% ± 15.82% for the Gaussian and 21.27% ± 15.54% for the modified Gaussian approach. The results for each component are listed in Table 2. 

It should be noted that the ICM measurements might be sensitive to the gradient. Some gradients, more specific some modifier, are UV/VIS active itself. Even if the gradient does not become visible in the chromatogram at a specific wavelength, there might be an effect to the measurement. When the calibration is done with chromatographic runs of the single components as described, most of this problem is already solved since the gradient is already present in the calibration measurements. Otherwise, a blank run should be made before the measurements. The UV data measured during the actual run are than corrected by subtracting the blank run UV/VIS data.

**Figure 4:**
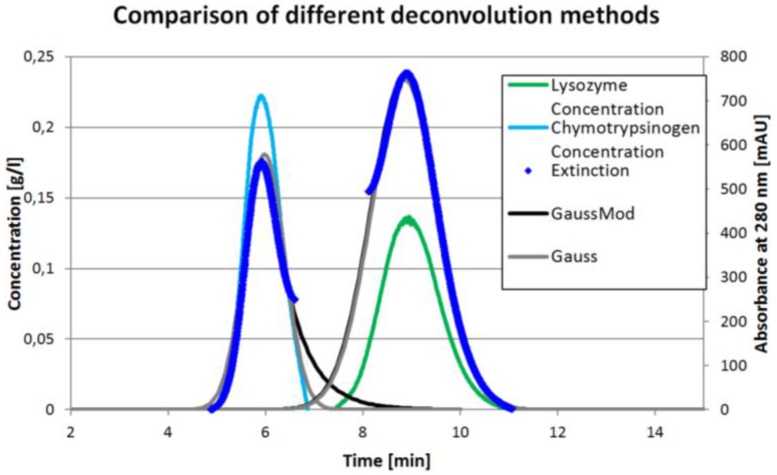
Comparison of the different peak deconvolution methods.

Figure 4 shows the course of the different peak deconvolution methods. The peak in the middle is left out for clarity. It can be seen that the perpendicular area determination is always neglecting some area. On the other hand, both Gaussian functions (grey and light grey) overestimate the real behavior. It can be seen that the mathematical fits show the first and third peak to be overlapping, which is not the case in reality. The ICM method represents the real behavior best. Because it is not based on some model assumptions, the ICM method further has the potential to also identify tag along effects.

The mathematical methods can only be applied after the run or with huge delay, since the peak needs to be fully developed or at least needs to be developed past the maximum. The ICM measurements presented above were performed inline and with 100 ms between each data point. Since the self-written program is capable of communication with PLC, the data can be used to do live pooling or process optimization during a production run.

The results above were produced with extinction coefficients known beforehand. Similar results could be obtained without knowing the coefficients beforehand. An analysis of the complete run can calculate the extinction coefficients and deconvolute the overlapping peaks by assuming pure components at the beginning and end of the first or last peak respectively. The coefficients for the second component can be calculated by knowing the first and second component coefficients and the total spectra. This would deconvolute the peaks, but not give the exact concentration, since the link between concentration, extinction coefficients and the spectra is missing. In combination with known extinction coefficients for known components, this approach can be used to detect unknown components and give their concentration as a pseudo concentration linked to a known component.

### 3.2. Immunoglobulin G (IgG) Monomer and Dimer with SIMPLS

**Figure 5:**
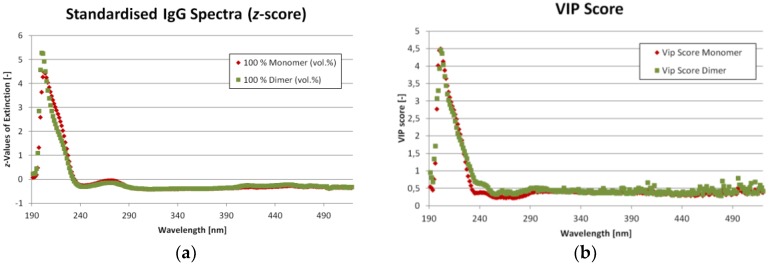
(**a**) Ultraviolet/Visible light (UV/VIS) spectra of the trainings data with the highest and lowest Immunoglobulin G (IgG) monomer content. The red dots represent the mixture with 100 vol.% monomer, the green dots with 100 vol.% dimer, respectively. However, this does not mean pure monomer or dimer since a conversion from one into the other takes place; (**b**) Variable Importance on Projections (VIP) scores for monomer (red) and dimer (green).

**Figure 6:**
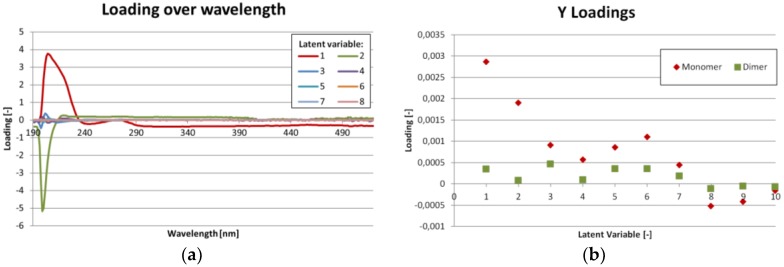
(**a**) Loading over wavelength diagram for the first eight latent variables. The higher the absolute value, the more important is this wavelength for the latent variable; (**b**) loadings of the Y results over latent variables. The higher the absolute value, the higher is the importance of this latent variable for the concentration calculation of monomer or dimer respectively.

As mentioned before, eleven different ratios of IgG monomer and dimer were measured from which nine were used for the SIMPLS calibration and three for the validation. The corresponding UV/VIS spectra for the mixture with the highest and lowest monomer content are shown in Figure 5a. The spectra are standardized (*z*-score) to emphasize the differences. One statistic model was created to calculate both IgG monomer and dimer concentration at the same time as opposed to creating two models, one for each component. The Variable Importance on Projections (VIP) can be found in Figure 5b. Both Figure 5a,b show, that there are only minor differences in UV/VIS absorption for IgG monomer and dimer. The most important region is from 200 to 290 nm. This predication is supported by Figure 6a, which shows the loadings for each latent variable over wavelength. Again, the highest values are in the region from 200 to 290 nm. The region beyond 390 nm might be dominated by noise. Figure 6b shows the importance of each latent variable for the concentration results (Y-matrix). As expected, the first latent variables are the most important. But especially for the dimer concentration, variables 5 to 7 also show significant contribution. 

As expected, the percentage of explained variance in the response increases with the number of latent variables used in the algorithm. For three latent variables, the percentage of explanation is 80%, for six variables it is over 93%. It maxes out at 98% with eight or more latent variables. Thus, eight latent variables where used for the validation measurements. The root mean square error of calibration (RSMEC) for eight latent variables is 0.14 mg/L for the IgG monomer and 0.025 mg/L for IgG dimer.

**Figure 7:**
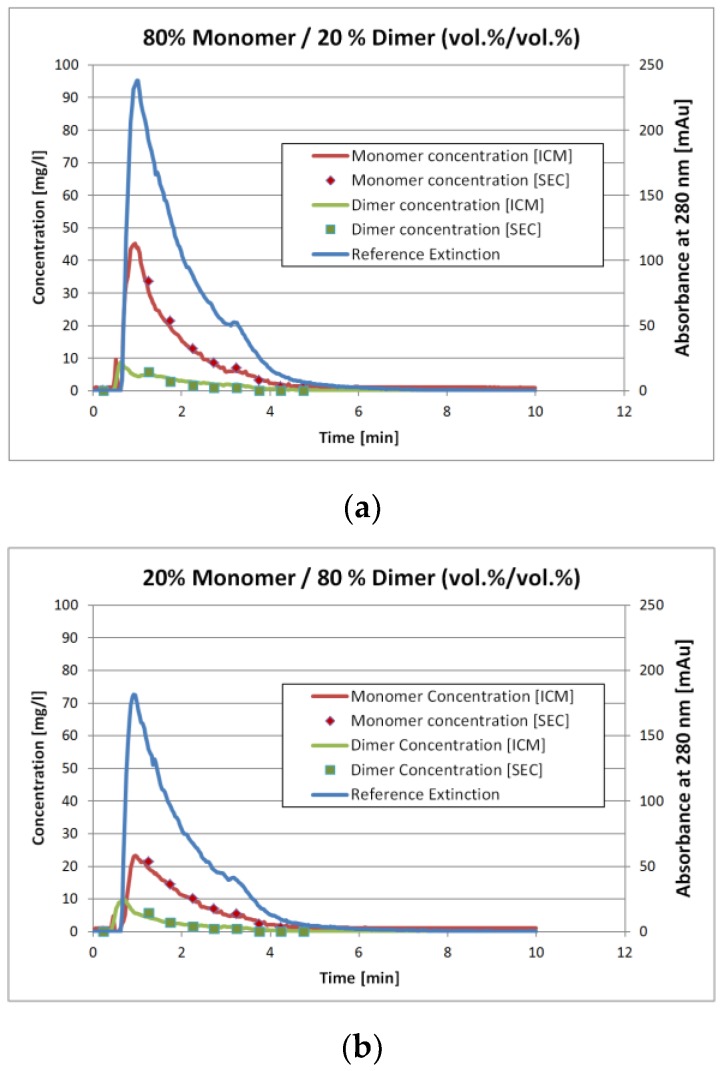
Concentration measurements over time of IgG monomer and dimer after injecting into a continuous stirred-tank reactor: (**a**) Volumetric mixing ratio of 80% monomer and 20% dimer; (**b**) Volumetric mixing ratio of 20% monomer and 80% dimer.

Figure 7a and Figure 4b show the results for two of the validation measurements. These experiments represent the injection of the sample with 80 vol.% monomer/20 vol.% dimer (Figure 4a) and 20 vol.% monomer/80 vol.% dimer (Figure 4b), respectively. Again, these numbers represent the volumetric mixing ratio of the SEC fractions as described earlier. Due to conversion of either monomer to dimer or vice versa the mixing ratio does not match the concentration ratio. 

The solid lines represent the ICM measurements whereas the squares with the same color represent the corresponding SEC analysis. The later are done by fractionation the stirred tank outlet with an interval of 0.5 min. The ICM measurements however are continuous. To compare the results, the ICM values for the 0.5 min interval were not averaged. Instead, the ICM concentration value for the corresponding time at the middle of the fractionation interval was used.

**Figure 8:**
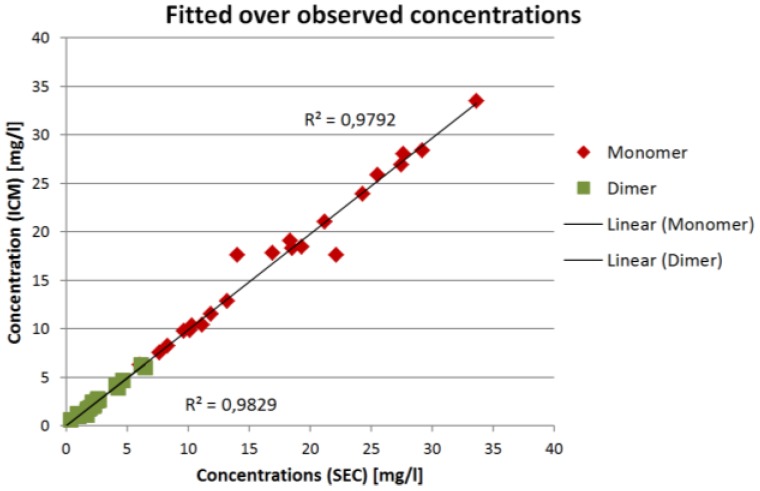
Concentrations based on fractionation and size exclusion chromatography (SEC) analyses compared to concentrations measured with inline concentration measurements (ICM).

The comparison for all validation experiments is shown in Figure 8:

The results in Figure 7 and Figure 8 show that the ICM measurements are in good agreement with the SEC measurements. The overall coefficient of determination *R*² is 0.98 for both IgG monomer and dimer. The root mean square error of prediction (RMSEP) is 0.13 mg/L for the IgG monomer and 0.019 mg/L for IgG dimer. 

The ICM measurements provide the concentrations and therefore composition of the outlet stream. This can be used to calculate the purity of the product stream in almost real time (100 ms). Purity calculation based on ICM measurements compared to the corresponding offline SEC gives a mean derivation of 0.15%. Within the 24 data points, the minimum deviation was 0.01% and the maximum deviation was 6%. 

Since the SIMPLS-algorithm reduces the UV/VIS spectra to a statistic model, it is possible that it only finds a certain fixed distribution between the two components. This would presumably give the same multiplier between both concentrations. Figure 4a,b however show that this is not the case. The extinction values for the 20 vol.% monomer run are roughly 25% lower than for the 80 vol.% monomer run. The IgG monomer concentration however is more than 50% lower and the dimer concentration is 15% higher. Hence, it is reasonable to assume that the algorithm determines the concentrations correctly instead of just splitting the extinction with a fixed proportion.

Since the test mixture was prepared with IgG samples after Protein A and SEC, the purity of the IgG was relatively high. For in-process measurements, for example after protein A, more components and impurities have to be taken into account. Depending on the impurities, the distinction between the IgG monomer and dimer might become more challenging. However, it is hypothesized that at least the distinction between IgG in total and other impurities should be possible. 

## 4. Conclusions

The proposed inline concentration measurement based on UV/VIS spectral data is a new and comparatively easy method for the real time quantification of UV/VIS active components. This indicates a huge potential for analytical as well as preparative and production-scale chromatography as well as for other unit operations. It is a key-enabling methodology for process development by aid of process modelling in order to determine model parameters efficiently—which is currently the major obstacle preventing a general use of simulation instead of empirics in industry for piloting and operation of almost any unit operation in regulated industry under Process Analytical Technology (PAT) and QbD design methods.

The inherent problem of overlapping peaks in analytical chromatography is reduced significantly by applying ICM. The accuracy of the quantitative determination of proteins could be shown to be improved to more than 99% compared to under 85% for conventional, state-of-the-art techniques.

The analysis time is less than 100 ms, so that the rate-limiting step is the data acquisition done by the detector itself. This enables a variety of process control options like live pooling and online optimization of the chromatographic process.

In the case of preparative separations, even for very similar components the purity can be measured at approximately 99.8% accuracy. This is an enormous advantage, since the real-time concentrations and purities are completely unknown for current process analytics. This leads to new possibilities for process control strategies. For example, batch chromatography could perform real-time pooling decisions based on the ICM data, leading to less batch failure and a higher product quality. Since product-related purity data are measured inline, there is a huge potential for cost and time savings by reducing offline analytics. The derived method overcomes obstacles in industrial application resulting from the dependency of extinction coefficients on detector type, age and signal strength by adopting reliable self-adjusting methods based on optimization algorithms, which provide mathematically proven quantitative reliability.

New process control options of multicolumn chromatography processes like Simulated Moving Bed (SMB) and their derivatives, e.g., integrated counter current chromatography (see [34]) based on the ICM-approach will be presented in the near future.

## Figures and Tables

**Table 1:** Deviation from single component to mixture measurement.

Protein	Mean Residence Time	
	Single Component	Mixture
	(min)	(min)
Chymotrypsinogene-A	6.07	5.96
Cytochrome-C	7.62	7.63
Lysozyme	9.03	9.01

**Table 2:** Deviation from single component to mixture measurement.

Protein	ICM	Perpendicular	Gaussian	Modified Gaussian
	(%)	(%)	(%)	(%)
Chymotrypsinogen-A	−0.63	−7.09	9.92	12.71
Cytochrom-C	0.50	−22.37	3.21	8.02
Lysozyme	0.19	−21.52	39.62	43.09
Mean Value	0.02	−16.99	17.58	21.27
Standard deviation	0.48	7.01	15.82	15.54
